# Oral Cholera Vaccine Development and Use in Vietnam

**DOI:** 10.1371/journal.pmed.1001712

**Published:** 2014-09-02

**Authors:** Dang Duc Anh, Anna Lena Lopez, Hung Thi Mai Tran, Nguyen Van Cuong, Vu Dinh Thiem, Mohammad Ali, Jacqueline L. Deen, Lorenz von Seidlein, David A. Sack

**Affiliations:** 1 National Institute of Hygiene and Epidemiology, Hanoi, Vietnam; 2 University of the Philippines Manila-National Institutes of Health, Manila, Philippines; 3 National Expanded Programme on Immunization, Hanoi, Vietnam; 4 Department of International Health, Johns Hopkins Bloomberg School of Public Health, Baltimore, Maryland, United States of America; 5 Menzies School of Health Research, Casuarina, Northern Territory, Australia

## Abstract

Anna Lena Lopez and colleagues give an overview of the cholera situation in Vietnam and discuss how an oral cholera vaccine was developed and used as a component of a public health strategy against the disease.

*Please see later in the article for the Editors' Summary*

**Summary Points**

- Vietnam is the first and only country in the world to regularly use oral cholera vaccines (OCVs) in their cholera control program.
- From 1998 to 2012, more than 10.9 million doses of the locally produced OCV were deployed in the country through its public health system.
- We present an overview of cholera epidemiology in Vietnam and the development and deployment of the OCV.
- Since 1997, the number of cholera cases in Vietnam has declined, in association with increased OCV use as well as improvements in socioeconomic and water and sanitation conditions. It is not possible to establish the relative contributions of each of these to the reduction in cholera rates.
- Hue, the only province to use OCVs consistently every year, has not reported any cholera case since 2003.
- As WHO organizes a stockpile of OCV for use in emergencies and recommends the use of OCVs together with traditional means of control, the experience in Vietnam will be helpful to other at-risk countries as they look towards adopting the vaccine in their cholera control programs.

## Cholera: A Continuing Public Health Threat

The emergence of cholera in Haiti highlighted the difficulties in containing cholera outbreaks with only safe water, sanitation, hygiene, and appropriate case management. In less developed settings where cholera occurs, these basic needs are often not met or are rapidly overwhelmed during man-made or natural disasters. Prior to the Haitian outbreak, countries in Africa and Asia had borne most of the cholera burden, with an estimated 1.4 billion people at risk, 2.8 million cases, and 100,000 to 200,000 deaths occurring annually [1],[2]; however, because of difficulties in surveillance and differences in reporting systems, only 245,393 cases with 3,034 deaths were reported to the World Health Organization (WHO) in 2012 [1]. This figure does not include the large number of acute watery diarrhoea cases reported in Asia, of which a significant proportion is caused by *Vibrio cholerae*. As cholera continues to be a global public health problem, in 2011, the World Health Assembly called for an integrated and comprehensive approach to cholera control, including oral cholera vaccines (OCVs) [3].

OCVs have been available for more than 20 years, but public health use has been limited. Vietnam is the first and currently the only country in the world to use killed OCVs routinely in its public health program. This article describes the cholera problem in Vietnam and how an oral cholera vaccine was developed and used as a component of a public health strategy against the disease.

## Cholera in Vietnam

Cholera has been endemic in Vietnam since 1964, when *V. cholerae* O1 El Tor was first identified in the country. Vietnam uses the term “severe watery diarrhoea” for culture-confirmed cases of cholera or clinically diagnosed cholera during an outbreak. Reports of cholera are forwarded to and collated by the Epidemiology Unit of the National Institute of Hygiene and Epidemiology (NIHE) under the Ministry of Health (see Text S1 and Table S1).

From 1991 to 2001, reported cases of severe watery diarrhoea were highest in the South Central Coast, followed by the North Central Coast. During the same period, Hue province in the North Central Coast had the highest annual incidence rate of severe watery diarrhoea cases, with the majority of cases coming from its capital, Hue city (NIHE, unpublished). In a review of cholera epidemiology in Vietnam from 1991 to 2001 by Kelly-Hope et al., the average annual incidence for the whole country was 2.7 cases per 100,000 people [4].

**Figure 1:**
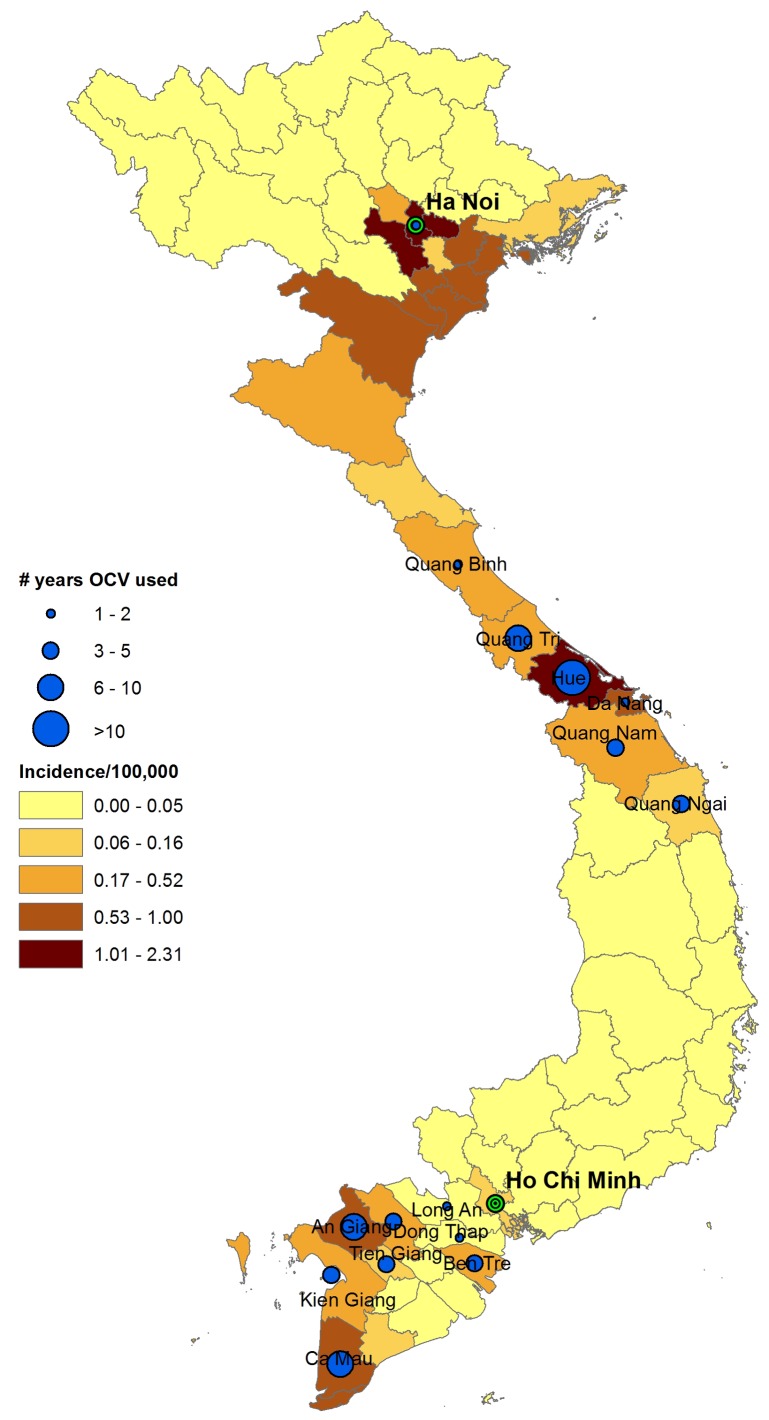
Map of average annual cholera incidence and oral cholera vaccine use, 1998–2012. Years when oral cholera vaccines were used: **An Giang** (1998, 2000, 2001, 2004, 2005, 2011); **Ben Tre** (2000, 2001, 2002, 2011); **Ca Mau** (1998, 2000, 2003, 2004, 2005, 2006, 2011); **Can Tho** (1999, 2000, 2002); **Da Nang** (2004, 2005); **Dong Thap** (2001, 2002, 2003); **Kien Giang** (1999, 2000, 2001, 2003); **Long An** (2001); **Quang Binh** (2006); **Quang Nam** (2004, 2005, 2006, 2007); **Quang Ngai** (2004, 2005, 2006); **Quang Tri** (1999, 2000, 2001, 2004, 2005, 2006, 2007); **Hue** (1998–2012); **Tien Gian** (2012); **Hanoi** (2008).

From 1992 to 1995, 2,500 to 6,000 cases were reported annually [5]; however, since 1997, a decline in the number of cases has been seen, including a 10-fold drop between 1996 and 1997. While this decrease may have been initially explained by the cyclical nature of the disease, the decline was sustained. From 1998 to 2006, the number of cases ranged from 0 to 343 annually, mostly in the southern provinces of An Giang, Ca Mau, and Dong Thap and the central provinces of Quang Tri and Hue (Figure 1). Between 1998 and 2002, only one other case was seen in Hue, until October 2003 when an outbreak with 200 cases was reported.

**Figure 2:**
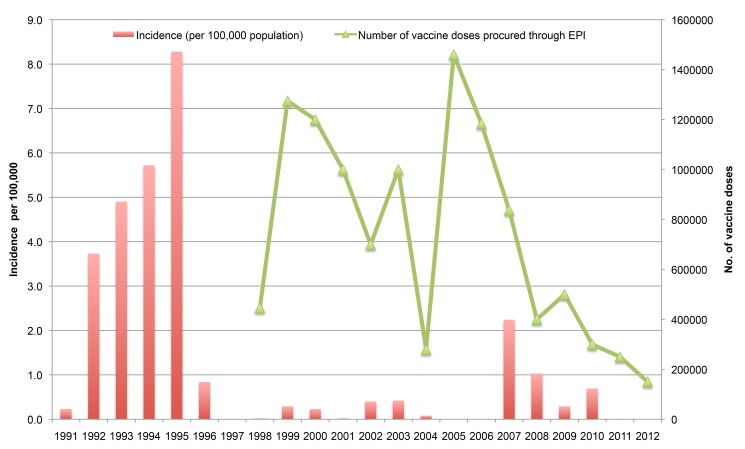
Incidence of cholera in Vietnam and number of vaccine doses procured through the National Expanded Programme on Immunization, 1998–2012. Number of vaccine doses procured through the EPI does not include the additional approximately 400,00 doses procured directly by the Ministry of Health for the 2008 mass campaign in Hanoi and the other doses for Long An and Vinh Long in 2001.

In October 2007, an increase in the number of cases was seen in Hanoi, subsequently affecting nearby provinces in the North. 1,907 and 886 cases were reported in 2007 and 2008, respectively. This outbreak in Hanoi was unusual because of the large number of cases that lasted through the winter season. Between 1998 and 2012, case numbers varied between 0 and 1,907 per annum, with an estimated annual incidence of 0 to 2.24 cases per 100,000 people (median 0.03 and mean 0.28) (Figure 2). The majority of the cholera cases were reported from the northern region during the 2007–2010 outbreaks.

The history of the development of oral cholera vaccine in Vietnam is shown in Table 1. The evolution in the formulation of the Vietnamese vaccine and how it is administered is shown in Table S2.

**Table 1:** History of the development of oral cholera vaccine in Vietnam.

Date	Events
1980s	Encouraged by trial results in Bangladesh of a vaccine containing killed whole-cells of *V. cholerae* O1 with the cholera toxin B subunit [25],[26], scientists from the Vietnam National Institute of Hygiene and Epidemiology developed their own vaccine, following technology transfer from Sweden [27]. The Vietnamese oral cholera vaccine was similar to the Swedish vaccine tested in Bangladesh, but did not contain the costly cholera toxin B subunit.
1992 to 1993	An open field trial was conducted in Hue city and showed that two doses of this killed OCV was safe, immunogenic, and provided 66% protection among individuals aged one year and older [28].
After 1992	Vietnamese scientists added *V. cholerae* O139 to the vaccine following reports of *V. cholerae* O139 outbreaks in India and Bangladesh. The resulting bivalent vaccine was shown to be safe and immunogenic [29].
1997	The bivalent vaccine was used in a large field trial in the city of Nha Trang, enrolling approximately 300,000 residents [30]. However, no cases of cholera were detected during the subsequent two years of follow-up precluding estimation of vaccine effectiveness (D. D. Anh, personal communication, 22 January 2014).
1997	The bivalent OCV was locally licensed as ORC-Vax, produced in Vietnam by the Company for Vaccine and Biological Production No.1 (Vabiotech), under the auspices of NIHE, and introduced in the country's routine vaccination programme.
1998	A large-scale mass vaccination program involving non-pregnant residents aged two years and older was conducted in half of the communes of Hue city. Intensive surveillance conducted for two years did not reveal any case of cholera.
2000	The remaining communes in Hue were vaccinated.
2003	A cholera outbreak occurred in Hue, allowing estimation of vaccine effectiveness. A case-control study estimated that the vaccine provided 50% protection three to five years after vaccination [6]. This is the first study to suggest that a killed OCV may provide long-term protection.
2009	ORC-Vax was reformulated to comply with cGMP and international standards and was licensed as mORC-Vax.

## Policy and Practice of Oral Cholera Vaccination in Vietnam

On 19 August 1997, the Vietnamese government issued a directive including OCVs in the National Expanded Programme on Immunization (EPI) schedule in areas at risk for cholera. In the EPI schedule, two doses of OCV are to be provided two weeks apart to children two to five years of age; however, local health policy makers may decide on the age groups included for vaccination depending on the local epidemiology, the number of vaccine doses available, and the capability of local government to support the vaccination.

From 1998 to 2012, more than 10.9 million doses of OCV were deployed in 16 provinces and major cities through Vietnam's EPI. Of these doses, 78% were procured from 1998 to 2006 (Figure 2) and were primarily used in the central and southern provinces where the incidence of cholera was higher. Figure 1 shows the provinces where OCVs were deployed. In addition, approximately 3 million doses were purchased from Vabiotech by the Ministry of Health (specifically during the 2007–2008 cholera outbreak), by the Asian Development Bank and by the private sector. Among the 16 provinces that have deployed OCVs, one province, the central coastal province of Hue, consistently used OCVs annually since 1998, providing more than 2.14 million doses to its inhabitants, or two doses of OCV per inhabitant (using 2010 population). Deployment of vaccines was made per commune, the lowest administrative unit.

In most provinces, vaccination targeted children aged two to five years. However, in Hue, all non-pregnant residents of communes aged two years and older were vaccinated every three to five years [6] depending on the local cholera epidemiology or occurrence of flooding. After the 2003 cholera outbreak in Hue, the provincial government provided vaccines to all eligible residents of the seven districts in a phased manner between 2004 and 2007. No cases were detected in Hue since 2003.

After Hue, Ca Mau province deployed the second highest number of vaccine doses, mostly in 2000–2001 and 2003–2006. In most years, Ca Mau provided vaccines to children aged two to five years. However, in 2001, 234 children at a primary school (aged five to 12 years old) in Ca Mau City received OCVs when the local health authorities determined that these children were at risk for cholera [7].

Quang Tri province, located just north of Hue near the Laotian border, immunized children aged two to five years from 2000 to 2007 (except in 2003) in communes perceived to be at risk. Cholera was reported in 1999 and 2000, then again in 2003. Because of its proximity to the border, OCVs were deployed when cases were reported in nearby Laos. Outbreaks of cholera were reported in Laos in 1994 to 1996 and then again in 1998 to 2002 [8]. No cholera case was reported in Quang Tri after 2004, despite the reported cholera outbreak in Laos in 2007 [9].

From 1998–2005, OCVs were deployed in select communes in An Giang province in the South for five years, where cholera was reported in five of the seven years. Cholera reappeared in An Giang in 2010, after five years of absence from 2005–2009, and OCVs were again deployed in 2011.

Cholera vaccines were used in areas with the highest number of cholera cases (Figure 1). Apart from Hue, An Giang and Ca Mau had the highest number of cases from 1998 to 2004 at 154 and 93 cases, respectively, during these years. However, apart from Hanoi, provinces that were hard-hit during the 2007–2008 outbreaks did not request nor did they deploy OCVs.

### Decision Making to Procurement

In the EPI, the decision to vaccinate a specific district is made at the provincial Centre for Preventive Medicine (CPM). Once cholera cases are detected in a district, the provincial CPM deliberates whether or not to vaccinate against cholera and to identify the age groups to be vaccinated. This decision has to be approved by the local provincial government, who finances the deployment of cholera vaccines at the local level.

Although provinces may request their preferred number of doses of OCV, procurement of vaccines is limited by the amount allocated for EPI vaccines in the national budget. If the number of OCV doses allocated to the province is less than the amount the province requested, then provinces may request additional doses for deployment the following year.

**Figure 3:**
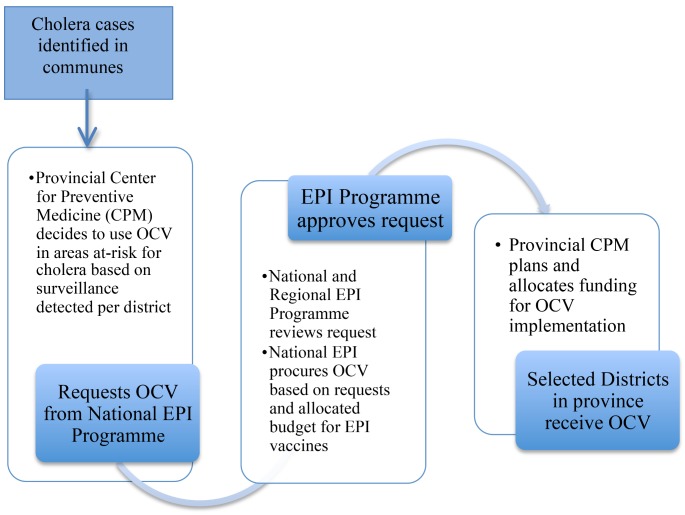
Process for oral cholera vaccine deployment.

While the National EPI pays for the purchase and shipping of OCVs, the provincial CPM is responsible for planning and implementing the vaccine deployment. Figure 3 shows the process, from OCV requisition to deployment. More recently, OCV procurement has been limited, and vaccines have to be procured for the following year. Requests must be made by October to receive vaccines the following year. Based on the government's current budget appropriation to the National EPI, doses are procured from Vabiotech, the manufacturer in Vietnam. This budget has gradually been reduced and the doses procured by the EPI have declined (Figure 2).

### Vaccine Deployment

Since 1998, OCVs are provided just prior to the expected cholera season, and in Hue the cholera season is during the months of May to November. Vaccines are delivered to the provincial CPM, then transported and stored at the district Health Centre. On the day of the campaign, vaccines are brought in cold boxes to commune health stations where vaccinations are conducted.

Vaccinations are arranged similarly to other mass campaigns. The vaccine is administered in commune health centres (CHC), where routine EPI vaccines are also given. Community mobilization consists of commune health workers going to households and informing them of the dates of vaccination and the need for two doses to ensure protection. During vaccination days, CHCs are open from 8A.M. to 5P.M., and campaigns usually last for two days, with one day being on a Saturday or a Sunday in order to allow for participation of those who work during the weekdays. Vaccination cards are provided to individuals who are vaccinated and logbooks containing the names of vaccine recipients are maintained. Those who were vaccinated are advised to return for the second round, scheduled two weeks later. Commune health workers provide reminders prior to the second round to ensure compliance with the schedule. If families are unable to go to the CHCs, commune health workers visit the residence to provide vaccines.

### Reactive Vaccinations

Reactive campaigns with OCVs are conducted once a cholera outbreak has already begun. Reactive vaccinations were conducted in Ca Mau city in 2001, in Hai Phong and Kien Giang in 2003, and in Hanoi in 2008. In Ca Mau, after three cases of *V. cholerae* O1 Inaba were identified, children attending two primary schools located near waterways were vaccinated [7]. During these years, OCVs were readily available from the National EPI, which allowed the immediate implementation of a mass vaccination campaign.

More recently, during the Hanoi outbreak in 2007–2008, the Ministry of Health directly purchased OCVs and, together with NIHE, implemented a mass vaccination campaign in two affected districts located near waterways where residents were assessed to have poor access to clean water, hygiene, and sanitation. The campaign included only individuals older than ten years (who were not pregnant) because the majority of the cases in Hanoi were in people aged ten years and older [10]. Organized by the Hanoi CPM and held in CHCs, the campaign lasted for three days per round. Vaccination cards were distributed and records were kept. Prior to the campaign, dissemination of information through radio, television, and commune health workers was conducted. Since the campaign was held in the middle of an ongoing outbreak, the interval between doses was only one week, instead of the usual two weeks.

### Monitoring of Vaccination

First and second dose recipients, vaccine coverage and wastage are reported to the National EPI. Although an incident of mass psychogenic illness among primary school children occurred in Ca Mau City in 2001 [9], no serious adverse events have been reported with OCVs since 1998. The small number of adverse events reported with OCVs prior to 2002 may be partly due to a weak adverse events following immunization (AEFI) monitoring system in Vietnam; however, improved AEFI monitoring was initiated in 2002 and expanded nationwide in 2008. A review of the AEFI surveillance in 2009 found a low rate of AEFI across the country [11].

### Costs of Vaccination

In 1998, costs for the first cholera vaccination in Hue City were calculated at US$0.89 per fully vaccinated person, and 79% of this amount was spent on vaccines [12]. More recent estimates of costs of vaccination were calculated from the 2013 campaign in Hue. The National EPI procured vaccine from the manufacturer at US$0.48 per dose (at 2012 exchange rate US$1 to VND 20,828) [13] and program cost to distribute and administer OCVs was US$0.11 per fully vaccinated person. Thus, about US$1.07 was spent per person vaccinated and 90% of this went to vaccine purchase. Because mass campaigns are held yearly in Hue and are part of the routine public health provision, implementation required minimal additional costs and are lower than might be expected if not integrated into a routine system. The program expenditures included costs for transport boxes, ice packs, printing of forms, vaccination cards, logbooks, and posters, training, and honoraria for the staff involved in the vaccination campaign. In 2013, the campaign immunized 46,398 two-dose vaccine recipients from five communes and utilized 130 health staff from the respective communes at a cost of approximately US$5,177. This amount did not include other costs routinely covered by the provincial EPI such as costs for social mobilization and waste management. Since cholera vaccination is now part of the public health program in Hue, it is difficult to attribute community health workers' visits for social mobilization and for waste management separately.

## Lessons Learnt

Vietnam has now delivered over 10 million doses of vaccine, and OCV use has been associated with a substantial decline in cholera in this country. Hue, a province that has used OCVs annually since 1998, has not reported any cholera cases since the last outbreak in 2003. Furthermore, quarterly environmental sampling of the water from the Perfume River running through Hue City has consistently been negative for *V. cholerae* since 2005 (D. D. Anh, personal communication, 22 January 2014). A factor influencing this decision to use OCVs in Hue was the burgeoning tourism sector; the local government of Hue understood that cholera outbreaks would hinder tourists from visiting their province and felt that continued use of OCVs, along with improvements in water and sanitation, was important to their cholera control effort.

While the absence of cholera may have been affected by economic progress and improvements in water and sanitation facilities in Hue, access to clean water and sanitation in the province has not been uniform. In 2009, a survey revealed that only 37% of the surveyed individuals in Hue had access to a centralized water system, lower than the reported 52%. Furthermore, this supply was intermittent through the day, especially during times of drought [14]. Nationwide, there are substantial differences among urban and rural households. Of urban homes, 58% have water piped in directly, while only 9% of rural homes had piped water at home. Access to improved sanitation increased from 46% in 1995 to 75% in 2011. Similarly, substantial gaps exist between urban and rural homes, with 93% and 67% having access to improved sanitation [15]. The Government of Vietnam and international donors have spent more than US$1 billion in improving water and sanitation [16] and expect to spend more money to address these gaps. There is no doubt that water supply programs and improved sanitation are advantageous for long-term control of various diseases including cholera; however, cost-benefit analysis has shown that in certain situations, community-wide vaccination programs may be more equitable in the short term [17].

The impact of OCVs is harder to assess in other provinces because of the variability in implementation. There was a precipitous decline in the number of cholera cases in 1997, even prior to extensive usage of OCVs. The initial decline may have been due to the cyclical nature of the disease. However, this decline was sustained, and apart from the outbreaks in Hanoi and the northern provinces in 2007, there were never more than 1,000 reported cases nationwide, annually. Furthermore, if the province followed the recommended age for oral cholera vaccination, which is two to five years old, the number of doses deployed would be lower. Recent studies have shown that OCVs confer herd protection provided that high enough coverage is achieved. In Kolkata, coverage of at least 28% resulted in indirect protection. If only two- to five-year-olds were vaccinated, indirect protection would have been less likely. In contrast, in Hue, where OCVs are given to all non-pregnant individuals older than one year, indirect protection of the unvaccinated segment of the population may explain the continued absence of cholera despite the phased vaccination that the province implements.

This assessment shows a temporal association between the sustained decline in cholera incidence and increased use of OCVs. It does not aim, nor is it able to show an overall causal relationship between OCV use and the general decline in cholera cases. Cholera control in the country may have been enhanced by factors other than vaccination, including implementation of public health control measures and improvement of water and sanitation infrastructure, as well as general economic development. Instead, the experience in Vietnam shows how OCVs have been used as an immediate measure against cholera while appropriate infrastructure is built, and how they have been included as part of an integrated strategy to control cholera.

Vietnam uses a “bottom-up” approach in determining use of OCV. The provincial CPM determines how many doses are to be requested and how to deploy them. Depending on the national budget and the availability of vaccine, vaccine is provided to the provinces. The vaccine is then provided without cost to the province, but the province must pay for the cost of its distribution and administration. This method facilitates local decision making and places additional responsibility on the local authorities to evaluate their needs and to cover the programmatic costs for vaccination. This approach assumes that local authorities have information on how best to determine if they need vaccine and how to allocate it within the province.

A factor that may have affected the non-deployment of OCVs, especially during the 2007–2008 outbreaks, may be the expenditures that provinces have to bear in mounting vaccination campaigns. In Hue, the incremental costs for mounting the campaign may not be substantial; however, in places that do not routinely conduct mass cholera vaccination, the costs may be higher. Since cholera cases will more likely affect these less developed areas, allocations to support program costs for OCV campaigns may be needed to prevent cholera in these high-risk areas since improvements in water and sanitation will take time.

OCV continues to be used in Vietnam; however, budgetary constraints have resulted in fewer doses being procured by the government, due to the perception of a lower threat from cholera as well as other competing priorities. As vaccines for other diseases are being recommended, governments will prioritize which vaccines to implement. With the need to prioritize, cholera surveillance becomes increasingly important to understand when and where to implement future OCV campaigns.

Though endemic cholera has declined in Vietnam, cholera may also spread from other countries. The last large cholera outbreak in Hanoi and other northern provinces lasted for 20 months, affecting more than 1,500 individuals in 22 cities and provinces of northern Vietnam. Patient isolates from this outbreak were similar to *V. cholerae* O1 isolates obtained during the cholera outbreaks in Thailand in January to October 2007 and in Laos in December 2007 [18]. These isolates had not been previously identified in Vietnam, suggesting recent importation [19]. Vulnerability to such cholera outbreaks emphasizes the need for continued vigilance by enhancing surveillance and improving control.

## Future Use of Oral Cholera Vaccines

As the cholera incidence in Vietnam decreases, the most affected age groups will change. Adults and older children will increasingly bear the brunt of the disease. Indeed, in the 2007–2009 outbreak in northern Vietnam, among 8,064 cases only 4.6% of cholera cases were reported among children aged more than five years. Intensified disease surveillance should be conducted to monitor these epidemiologic changes to guide the government in future policies as regards OCV use. Furthermore, as the economy of Vietnam improves, initiatives toward cholera elimination may be considered.

Since Vietnam is the only country that has used OCVs extensively as part of an overall national strategy to control cholera, the experience with this vaccine in Vietnam may be useful for other areas now considering its use. The revised recommendations of the WHO encouraging the use of OCV in endemic areas and the decision by Global Alliance for Vaccines and Immunization (GAVI) to help support the use of OCVs should lead to increased use of the vaccine. In July 2013, a global cholera vaccine stockpile was developed as an additional tool for cholera control. The vaccine currently included in the stockpile is Shanchol, an OCV very similar to mORC-Vax. Shanchol was shown to be effective [20] and feasible to use in various settings [21]–[23], and models have shown that more cases were prevented when used in combination with Water, Sanitation, and Hygiene (WASH) [24]. The global stockpile requires a reliable supply of OCV; unfortunately, the OCV from Vietnam is currently not WHO-prequalified and therefore cannot be purchased by United Nations agencies. However, the National Regulatory Agencies (NRA) of other countries at risk could license mORC-Vax; and registration would allow use of mORC-Vax in their countries. Vabiotech, together with the Vietnamese NRA, is working to have the vaccine prequalified.

This experience of Vietnam in deploying a two-dose oral cholera vaccine for more than 15 years indicates that the vaccine can be feasibly used in public health settings. Some of the lessons from Vietnam may prove to be useful in other countries where cholera remains a problem. The availability of a vaccine that may be used, even during an outbreak as part of an integrated strategy provides hope in countries that continue to battle the scourge of this disease.

## Supporting Information

Table S1 Immunization schedule in Vietnam's expanded programme on immunization.
(DOCX)

Table S2 Changes in formulation of the Vietnamese oral cholera vaccine.
(DOCX)

Text S1 Background information and data sources.
(DOCX)

## Abbreviations

AEFI: adverse events following immunization
CHC: commune health centres
CPM: Centre for Preventive Medicine
EPI: Expanded Programme on Immunization
GAVI: Global Alliance for Vaccines and Immunization
NIHE: National Institute of Hygiene and Epidemiology
NRA: National Regulatory Agencies
OCV: oral cholera vaccine
WASH: Water, Sanitation, and Hygiene
WHO: World Health Organization
