# Supplementary material for: Oral Cholera Vaccine Development and Use in Vietnam
Source: PLoS Med. 2014 Sep 2;11(9):e1001712. doi: 10.1371/journal.pmed.1001712 (PMC4151976; doi:10.1371/journal.pmed.1001712)
Supplement: Table S1 — Immunization schedule in Vietnam's expanded programme on immunization. (DOCX) [file pmed.1001712.s001.docx]

**Supporting Information: Table S1. Immunization Schedule in Vietnam’s Expanded Programme on Immunization**

| Vaccine | Schedule | Comments |
| --- | --- | --- |
| BCG | Birth |  |
| Hep B | Birth |  |
| DTwP-Hib-HepB | 2, 3, 4 months |  |
| OPV | 2, 3, 4 months |  |
| Measles | 9 , 18 months |  |
| Japanese encephalitis | 12 months; + 2 weeks; 2 years; | High risk areas |
| Cholera | 2-5 years | High risk areas |
| Typhoid | 3 years | High risk areas |
