# Supplementary material for: Oral Cholera Vaccine Development and Use in Vietnam
Source: PLoS Med. 2014 Sep 2;11(9):e1001712. doi: 10.1371/journal.pmed.1001712 (PMC4151976; doi:10.1371/journal.pmed.1001712)
Supplement: Table S2 — Changes in formulation of the Vietnamese oral cholera vaccine. (DOCX) [file pmed.1001712.s002.docx]

**Supporting Information: Table S2. Changes in formulation of the Vietnamese oral cholera vaccine.**

| **Vaccine strain** | **Formulation 1 (1992)** | **Formulation 2 (1997)** | **Formulation 3 (2000)** | **Formulation 4 (2009)** |
| --- | --- | --- | --- | --- |
| *V. cholerae* O1 Inaba El Tor strain Phil 6973 formalin killed | 2.5 x 10^10^ cells | 5 x 10^10^ cells | 5 x 10^10^ cells | 600 Elisa units (EU) of lipopolysaccharide (LPS) |
| *V. cholerae* O1 Ogawa classical strain Cairo 50 heat killed | 2.5 x 10^10^ cells | 2.5 x 10^10^ cells | 2.5 x 10^10^ cells | 300 EU LPS |
| *V. cholerae* O1 Ogawa classical strain Cairo 50 formalin killed | -- | -- | -- | 300 EU LPS |
| *V. cholerae* O1 Inaba classical strain 569B formalin killed | 2.5 x 10^10^ cells | 2.5 x 10^10^ cells | 2.5 x 10^10^ cells | -- |
| *V. cholerae* O1 Inaba classical strain Cairo 48 heat killed | 2.5 x 10^10^ cells | 2.5 x 10^10^ cells | -- | 300 EU LPS |
| *V. cholerae* O139 strain 4260B formalin killed | --- | 5 x 10^10^ cells | 5 x 10^10^ cells | 600 EU LPS |
| Licensure | -- | Locally licensed as ORC-Vax | Locally licensed as ORC-Vax | Locally licensed as mORC-Vax® |
