# Supplementary material for: Oral Cholera Vaccine Development and Use in Vietnam
Source: PLoS Med. 2014 Sep 2;11(9):e1001712. doi: 10.1371/journal.pmed.1001712 (PMC4151976; doi:10.1371/journal.pmed.1001712)
Supplement: Text S1 — Background information and data sources. (DOCX) [file pmed.1001712.s003.docx]

**Supporting Information: Text S1**

**Background Information and Data Sources**

*Vietnam: the country and its public health system*

The Socialist Republic of Vietnam is a rapidly developing, lower middle-income country located in the Indochina peninsula. The country is bordered by the South China Sea to the east, Laos and Cambodia to the west and China to the north. With an area of 331,051.4 km^2^**,** it has 88.78 million inhabitants, making it one of the most densely populated countries in the world [1]. Residents are distributed in four geographic regions – the North, South, Central and Highland regions encompassing 63 provinces and autonomous cities. The terrain is diverse with mountainous northwestern and central highlands and extensive low-lying areas in the Red River delta and the Mekong delta. Vietnam has a tropical climate in the south, monsoonal in the north with hot, rainy season (May to September) and warm, dry season (October to March) [2]. Worldwide, Vietnam ranks fourth in terms of number of people exposed to floods, with excess rainfall occurring particularly in the North central and South-central coastal regions. In addition, with more than 2,360 rivers and streams longer than 10 km, riverine flooding also occurs frequently [3].

The Ministry of Health (MOH) oversees Vietnam’s public health system. Under the MOH are four regional institutes covering the 63 provincial Centres for Preventive Medicine (CPM) in the Northern, Central, Southern and Central Highland provinces. The NIHE oversees the Northern provinces and houses the national EPI programme. Under each provincial CPM are district CPMs that supervise the commune health centres, the primary level in the health system. Table S1 shows the EPI vaccine schedule.

Vietnam’s health indicators have steadily improved since the 1980s, with infant mortality and under 5 mortality rates at 15.5 and 23.3, per 1,000 live births, respectively, in 2011 [4]. Access to improved water sources has been increasing from 67% in 1995 to 96% in 2011; however, there are substantial differences among urban and rural households. 58% of urban homes have water piped in directly, while only 9% of rural homes had piped water at home. Access to improved sanitation increased from 46% in 1995 to 75% in 2011. Similarly, substantial gaps exist between urban and rural homes with 93% and 67% having access to improved sanitation [5].

*Surveillance for cholera*

The provincial CPMs have the capability to isolate *V. cholerae* and identify cases early on. Cases of cholera were routinely collected by CPMs at the district level as part of the National Surveillance System. Culture identification is available at the provincial CPM level and isolates were submitted to NIHE for confirmation.

*Assessment of oral cholera vaccine (OCV) implementation*

To understand Vietnam’s OCV implementation, we explored OCV implementation using national, local EPI data as well as reports from 1998 to 2012. We assessed cholera epidemiologic patterns across the country from 1998 to 2012 using data from NIHE. Annual OCV use as reported by provincial CPM to the national EPI programme was obtained from Annual EPI Reports. Since not all vaccines were procured through the national EPI, additional data was obtained from government reports and Vabiotech as regards the number of doses procured directly by the Ministry of Health or other agencies.

The implementation of OCV vaccination programmes relies on local staff. To understand OCV implementation at the local level, interviews with the Hue EPI programme manager were conducted. Program monitoring, costs and number of staff required for implementation were obtained locally from Hue, since Hue has been known to use OCV annually.

*Assessment of cholera epidemiology*

Data on cholera cases occurring from 1998 to 2012 were obtained from the Epidemiology Department of NIHE. Cases of cholera were routinely collected by CPMs at the district level as part of the National Surveillance System. Culture identification is available at the provincial CPM level and isolates were submitted to NIHE for confirmation. Data were based on treated episodes of cholera. To ensure completeness, a review of published literature using the search terms “Vietnam” and “cholera” limited by the years 1998 to 2012 was conducted. Data from NIHE were compared with published reports from Kelly-Hope et al, which analysed data from 1991 to 2001. Cholera data were mapped with provinces that have deployed OCV.

*Vietnamese oral cholera vaccine*

Table S2 shows the evolution of the locally produced OCV. ORC-Vax® and mORC-Vax® used in the EPI Programme are packaged in 7.5 ml multi-dose vials containing 5 doses of 1.5 ml each. During administration, each dose is aspirated from the vial using a needle-less syringe and squirted directly to the mouth of each vaccinee. Vaccines are stored at 2-8**°**C.

**References**

1. (2014) Vietnam: Country Data. Available from [http://data.worldbank.org/country/vietnam - cp_wdi](http://data.worldbank.org/country/vietnam#cp_wdi). Accessed: 26 January 2014. The World Bank.

2. (2014) CIA Library: The World Factbook -Vietnam. Availablfe from: https://<http://www.cia.gov/library/publications/the-world-factbook/geos/vm.html>. Accessed on 26 January 2014.

3. Thomas T, Christiaensen L, Do QT, Trung LD (2010) Natural Disasters and Household Welfare: Evidence from Vietnam. The World Bank.

4. (2013) General Statistics Office of Vietnam. Available from <http://www.gso.gov.vn/default_en.aspx?tabid=467&idmid=3>. Accessed on 26 January 2014.

5. JMP WHO / UNICEF Joint Monitoring Programme (JMP) for Water Supply and Sanitation. Country file: Vietnam.
